# Supplementary material for: Asymmetric ON-OFF processing of visual motion cancels variability induced by the structure of natural scenes
Source: eLife. 2019 Oct 15;8:e47579. doi: 10.7554/eLife.47579 (PMC6884396; doi:10.7554/eLife.47579)
Supplement: Transparent reporting form [file elife-47579-transrepform.docx]

***eLife’s* transparent reporting form**

We encourage authors to provide detailed information *within their submission* to facilitate the interpretation and replication of experiments. Authors can upload supporting documentation to indicate the use of appropriate reporting guidelines for health-related research (see [EQUATOR Network](http://www.equator-network.org/%20)), life science research (see the [BioSharing Information Resource](https://biosharing.org/)), or the [ARRIVE guidelines](http://www.plosbiology.org/article/info:doi/10.1371/journal.pbio.1000412) for reporting work involving animal research. Where applicable, authors should refer to any relevant reporting standards documents in this form.

If you have any questions, please consult our Journal Policies and/or contact us: [editorial@elifesciences.org](mailto:editorial@elifesciences.org).

**Sample-size estimation**

- You should state whether an appropriate sample size was computed when the study was being designed
- You should state the statistical method of sample size computation and any required assumptions
- If no explicit power analysis was used, you should describe how you decided what sample (replicate) size (number) to use

Please outline where this information can be found within the submission (e.g., sections or figure legends), or explain why this information doesn’t apply to your submission:

In behavioral experiments, we used 40 flies to extract kernels. This is comparable to a previous study (Clark et al., 2011) where 48 flies were used in a similar experiment. Each fly went through two visual stimuli. We used 10-20 flies (**Method**, **Supplementary Table 1**) to measure third-order glider response. This is comparable to a previous study (Clark et al., 2014) where 11 flies are used in a similar experiment.

In simulations (**Method**, **Supplementary Table 2**), for each condition, we run 10 independent simulations, and each simulation contains 800-1000 independent trials. In the pilot experiment, we tested HRC’s performance with 1000 trials, and found the performance was stable with independent instantiations of 1000 trials. We used the same sample size for all later simulations.

**Replicates**

- You should report how often each experiment was performed
- You should include a definition of biological versus technical replication
- The data obtained should be provided and sufficient information should be provided to indicate the number of independent biological and/or technical replicates
- If you encountered any outliers, you should describe how these were handled
- Criteria for exclusion/inclusion of data should be clearly stated
- High-throughput sequence data should be uploaded before submission, with a private link for reviewers provided (these are available from both GEO and ArrayExpress)

Please outline where this information can be found within the submission (e.g., sections or figure legends), or explain why this information doesn’t apply to your submission:

The raw data of behavioral experiment can be found in Dryad Digital Repository.

We excluded a fly if the fly did not walk, did not turn, or had an extreme bias in turn direction, as described in a previous study (Creamer et al. 2019). Each fly contributed a single measurement to the kernel estimates.

**Statistical reporting**

- Statistical analysis methods should be described and justified
- Raw data should be presented in figures whenever informative to do so (typically when N per group is less than 10)
- For each experiment, you should identify the statistical tests used, exact values of N, definitions of center, methods of multiple test correction, and dispersion and precision measures (e.g., mean, median, SD, SEM, confidence intervals; and, for the major substantive results, a measure of effect size (e.g., Pearson's r, Cohen's d)
- Report exact p-values wherever possible alongside the summary statistics and 95% confidence intervals. These should be reported for all key questions and not only when the p-value is less than 0.05.

Please outline where this information can be found within the submission (e.g., sections or figure legends), or explain why this information doesn’t apply to your submission:

The statistical analysis for extracted kernels can be found in figure legends of **Figure 4.C** and Method section **Testing the significance of the measured third-order kernel with “null kernels”**. The statistical analysis for glider responses can be found in **Supplementary Table 1.**

The statistical analysis for simulations can be found in Method, **Evaluating the performance of a motion detector**, and **Evaluating the improvement added by the third-order response.**

(For large datasets, or papers with a very large number of statistical tests, you may upload a single table file with tests, Ns, etc., with reference to sections in the manuscript.)

**Group allocation**

- Indicate how samples were allocated into experimental groups (in the case of clinical studies, please specify allocation to treatment method); if randomization was used, please also state if restricted randomization was applied
- Indicate if masking was used during group allocation, data collection and/or data analysis

Please outline where this information can be found within the submission (e.g., sections or figure legends), or explain why this information doesn’t apply to your submission:

This does not apply our submission. We did not allocate samples into experimental groups.

**Additional data files (“source data”)**

- We encourage you to upload relevant additional data files, such as numerical data that are represented as a graph in a figure, or as a summary table
- Where provided, these should be in the most useful format, and they can be uploaded as “Source data” files linked to a main figure or table
- Include model definition files including the full list of parameters used
- Include code used for data analysis (e.g., R, MatLab)
- Avoid stating that data files are “available upon request”

Please indicate the figures or tables for which source data files have been provided:

We provided the sourced data for glider response data (**Figure 4.D, Supplementary Figure 4-1.B**) and the extracted second- and third-order kernels (**Figure 2.DE, Figure 4.BC**) in Dryad Digital Repository, and corresponding code in the repository provided below.

The parameters for HRC can be found in **HRC model** section in Methods. The parameters for contrast computation can be found in **Preprocessing photographs** section in Methods.

The analysis code, and the link to natural scene dataset can be found in this repository <https://github.com/ClarkLabCode/ThirdOrderKernelCode>.

Data is available to reviewers here:

<https://datadryad.org/review?doi=doi:10.5061/dryad.7jm87bt>
